# The Immunohistochemical Expression of the Serine and Arginine-Rich Splicing Factor 1 (SRSF1) Is a Predictive Factor of the Recurrence of Basal Cell Carcinoma: A Preliminary Study on a Series of 52 Cases

**DOI:** 10.3390/medicina58010139

**Published:** 2022-01-17

**Authors:** Giuseppe Broggi, Davide Barbagallo, Francesco Lacarrubba, Anna Elisa Verzì, Giuseppe Micali, Michele Purrello, Rosario Caltabiano

**Affiliations:** 1 Department of Medical, Surgical Sciences and Advanced Technologies “G.F. Ingrassia”, Anatomic Pathology, University of Catania, 95123 Catania, Italy; rosario.caltabiano@unict.it; 2 Department of Biomedical and Biotechnological Sciences, Section of Biology and Genetics “Giovanni Sichel”, University of Catania, 95123 Catania, Italy; dbarbaga@unict.it (D.B.); purrello@unict.it (M.P.); 3 Dermatology Clinic, University of Catania, 95123 Catania, Italy; francesco.lacarrubba@unict.it (F.L.); aeverzi@gmail.com (A.E.V.); giuseppe.micali@unict.it (G.M.)

**Keywords:** basal cell carcinoma, local recurrence, prognosis, SRSF1, immunohistochemical expression, predictive factor

## Abstract

*Background and Objectives*: Basal cell carcinomas (BCCs) are the most frequent skin tumors; although they usually exhibit a good prognosis, it has been reported that there is a 2–8% rate of local recurrence of surgically-excised BCCs, even in the presence of tumor-free surgical margins. Several histological and clinical risk factors have been associated with a higher risk of local relapse; however, the exact pathogenetic mechanisms that regulate the local recurrence of these tumors are still to be elucidated. The serine and arginine-rich splicing factor 1 (SRSF1) is an RNA-binding protein whose oncogenic function has been described in numerous forms of human cancers, including brain, lung, and prostate tumors. We evaluated the correlation between SRSF1 immunoexpression and the local recurrence of BCCs. *Materials and Methods*: Fifty-two cases of surgically excised BCCs with free-tumor margins (10 high-risk and 42 low-risk variants), for which follow-up data were available, were selected. Local recurrence occurred in only 5 cases. *Results*: We found high and low immunoexpressions of SRSF1 in 18 and 34 cases, respectively. A statistically significant association between high SRSF1 immunoexpression and the local recurrence of BCC was found (*p* = 0.0433). *Conclusions*: Our immunohistochemical results suggest an active role of SRSF1 in inducing a local recurrence of BCCs; however, further studies on a larger series are needed to validate our findings.

## 1. Introduction

Basal cell carcinoma (BCC) represents the most frequent cutaneous neoplasm of fair-skinned populations [1]; the biological behavior of BCC is classically characterized by slow growth, a rare local recurrence, and an extremely low metastatic potential [1]. As its pathogenesis has been mainly related to sun exposure, the incidence rates of BCCs vary in relation to geographical latitude and are higher in patients with Fitzpatrick skin phototypes I and II [1]. BCC is usually diagnosed by clinical examination and dermoscopy [2]; in recent years, further diagnostic techniques, such as in vivo reflectance confocal microscopy (RCM) and line-field optical computed tomography (OCT) have contributed to the improvement of the sensibility and specificity of the clinical diagnosis of both inflammatory and neoplastic skin diseases, including BCC [3,4,5,6,7]. Histologically, BCCs are composed of islands/nests of basaloid cells with scant cytoplasm, hyperchromatic nuclei, and peripheral nuclear palisading; neoplastic cells are often surrounded by a fibro-myxoid stroma that induces tumor retraction [8]. Although the therapeutic gold standard is the surgical excision with tumor-free histological margins, a percentage of surgically-excised BCCs tend to recur even in the presence of safe histological margins [9,10,11]. Numerous clinical and histological parameters have been classically associated with the recurrence of BCC [10,11]: (i) its localization, (ii) the tumor size, (iii) the histological subtypes, (iv) the histological evidence of an invasion of the reticular dermis and/or subcutis, and (v) the histological presence of a vascular/perineural invasion. Based on the histology, BCCs are distinguished into low-risk and high-risk subtypes [8]. The former includes the nodular, superficial, pigmented, infundibulo-cystic, and fibroepithelial variants; the latter includes the basosquamous, sclerosing/morphoeic, infiltrating, micronodular, and sarcomatoid variants. However, despite the above-mentioned predictive factors of local relapse, the reason why approximately 2–8% of surgically-excised BCCs with tumor-free margins locally recur at 5 years after surgery is still not clear [11]. Accordingly, it has been hypothesized that additional factors may contribute to the recurrence of this tumor.

The serine and arginine-rich splicing factor 1 (SRSF1) is an RNA-binding protein involved in both canonical and alternative mRNA splicing [12,13]. SRSF1 also exhibits several oncogenic functions, including the stimulation of angiogenesis, cell growth, and cell proliferation [12,13,14]. It has been found to be upregulated in numerous human malignancies, such as glioblastoma and breast carcinoma, as well as lung and colorectal cancer [12,13,14,15,16]. Recently, our research group also reported the poor prognostic role of SRSF1 in adult diffuse gliomas, prostate cancer, and mesotheliomas [17,18]. To the best of our knowledge, there are no studies about the expression of this protein in BCC to date.

The aim of the present study is to evaluate the potential relationship between SRSF1’s immunohistochemical expression and the recurrence of BCCs.

## 2. Materials and Methods

### 2.1. Ethics Statement and Sample Collection

Despite the compliance of the present study with the Helsinki Declaration, the non-interventional retrospective nature of our research did not require any informed consent by the local research ethics committee. All cases of surgically excised, histologically proven BCCs with tumor-free margins histological, diagnosed from 2016 to the present date, were retrieved from the Pathology files of the Department of Medical and Surgical Sciences and Advanced Technologies “G.F. Ingrassia” of the University of Catania. Among them, 52 cases, including hematoxylin and eosin-stained sections, paraffin-embedded blocks, and clinical data regarding the onset of local recurrence were selected. Clinico-pathological data were collected from the original pathologic reports. The following inclusion criteria were used: (i) paraffin-embedded blocks with enough tumor tissue to cut additional sections for immunohistochemistry had to be available, (ii) vital tumor tissue had to be contained within paraffin-embedded blocks, and (iii) tumor necrosis had to be absent or focal to preserve the immunoreactivity of the tissue.

### 2.2. Immunohistochemistry

A single representative paraffin-embedded block from each case was cut for immunohistochemical tests that were performed as previously reported [19]. Briefly, deparaffinized and pre-treated sections were incubated for 30 min at 37 °C with a mouse monoclonal anti-SRSF1 antibody (sc-33652, working dilution 1:50; Santa Cruz Biotechnology, Dallas, TX, USA). The evidence of brown chromogens inside the tumor nuclei were considered a positive immunohistochemical expression of SRSF1; as recommended on the antibody datasheet, we used unaffected gallbladder specimens as a positive control, while the omission of the primary antibody served as the negative control slides. All immunohistochemical slides were separately evaluated by two pathologists (G.B. and R.C.), removed of any clinico-pathological data, under a light microscope. A semi-quantitative assessment of the immunohistochemical findings was performed, as previously reported [17]. Briefly, a 0 to 3 score (weak, moderate, or strong) was established for the intensity of staining (IS), while a 0 to 4 scale (<5%, 5–30%, 31–50%, 51–75%, and >75%) for the extent score (ES). By multiplying the IS and ES, we obtained a 0 to 12 immunoreactivity score (IRS) of SRSF1 that was considered as a low score if ≤6 (L-IRS), and as a high score if >6 (H-IRS).

The Fisher exact test was performed to compare SRSF1 IRS values and occurrence of local relapse, while Pearson’s chi-squared test was used to correlate the SRSF1 IRS values with age, gender, the anatomic site, and the histological subtypes. A *p*-value of <0.05 was considered as statistically significant.

## 3. Results

### 3.1. Clinicopathologic Features of the BCC Cases from Our Series

All clinico-pathological and immunohistochemical features of the cases included in the present study are summarized in Table 1.

The study included 52 patients (32 males and 20 females) with an age ranging from 36 to 84 years (median age: 58 years). Tumors were located on the arms in 21/52 cases (40%), legs in 15/52 cases (29%), face in 10/52 cases (19%), shoulders in 5/52 cases (10%) and ankles in the remaining 1/52 case (2%).

Histologically, 10/52 cases (19%) were diagnosed as high-risk BCC variants (4/52 cases were basosquamous carcinomas, while 6/52 were sclerosing/morphoeic BCCs); the remaining 42/52 tumors (81%) exhibited a nodular and superficial BCC morphology in 27/52 (52%) and 15/52 cases (29%), respectively. All tumors had been surgically excised with tumor-free histological margins. The local recurrence of the disease occurred in only 5/52 cases (10%). Interestingly, 2 out of 5 (40%) locally recurrent tumors were high-risk histological subtypes (1 case of basosquamous carcinoma and 1 case of sclerosing/morphoeic BCC); the remaining 3 out of 5 (60%) relapsed BCCs were low-risk histological variants. All recurrent tumors exhibited the same histological subtype as the primary lesion.

### 3.2. SRSF1’s Immunohistochemical Expression and Its Relationship with the Local Recurrence of BCCs

**Figure 1:**
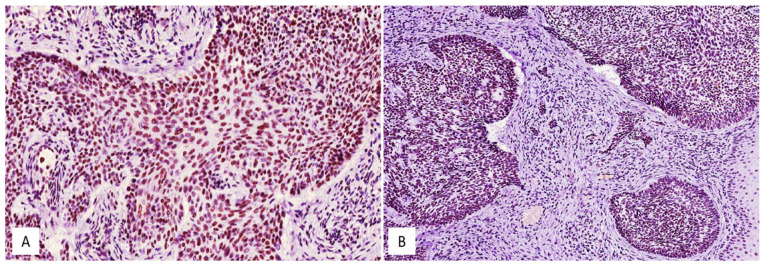
High (**A**) and low (**B**) immunohistochemical expression of SRSF1 in conventional basal cell carcinomas; immunoperoxidase, original magnifications 200× (**A**) and 150× (**B**).

**Figure 2:**
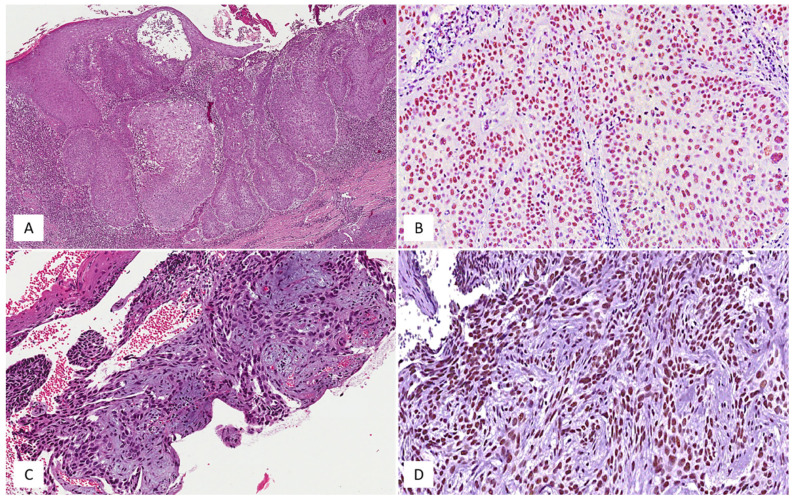
High-risk subtype of basal cell carcinoma. (**A**) Histological examination of basosquamous carcinoma showing nests and islands of basaloid cells with squamous differentiation; hematoxylin and eosin, original magnification 50×. (**B**) Neoplastic cells of basosquamous carcinoma are diffusely and strongly stained with SRSF1; hematoxylin and eosin, original magnification 200×. (**C**) Histological detail of a case of sclerosing/morphoeic basal cell carcinoma, exhibiting strands and confluent nests of basaloid cells, set in a dense fibrous stroma; hematoxylin and eosin, original magnification 200×. (**D**) The same case of sclerosing/morphoeic basal cell carcinoma is strongly and diffusely stained with SRSF1; hematoxylin and eosin, original magnification 250×.

High (H-IRS) and low (L-IRS) immunohistochemical levels of SRSF1 were found in 18/52 (35%) and in 34/52 (65%) cases, respectively (Figure 1 and Figure 2). Considering that 47/52 BCCs did not show local recurrence, only 14/47 (30%) exhibited a high immunohistochemical expression of SRSF1, while low immunostaining was observed in the remaining 33/47 cases (70%). Interestingly, 4 out of 5 (80%) locally recurrent BCCs exhibited a high SRSF1 immunohistochemical expression, while a low immunoexpression was found only in 1 out of 5 cases (20%). In this regard, the Fisher exact test showed a statistically significant correlation between a high SRSF1 immunohistochemical expression and its local recurrence (*p* = 0.0433) (Table 2). In addition, by correlating SRSF1 IRS with the other above-mentioned clinico-pathological parameters by Pearson’s chi-squared test, we found significant differences between SRSF1 IRS and the patient’s gender (*p* = 0.580), the tumor anatomic site (*p* = 0.031), and the histological subtype (*p* = 0.051) (Table 3, Table 4 and Table 5), while age was not associated with the SRSF1 immunoexpression (OR: 0.99, 95% CI 0.95–1.03, *p* = 0.84). In more detail, lower SRSF1 IRS values were associated with arm and leg sites and nodular histology. Conversely, a higher immunohistochemical expression of SRSF1 was found in superficial BCCs.

## 4. Discussion

Although BCC is classically considered a malignant tumor, it is characterized by slow growth and an extremely low metastatic potential [1,2]. A surgical excision with tumor-free histological margins is considered the treatment of choice [9,10,11]. However, it is estimated that 2–8% of radically-excised BCCs tend to locally recur after 5 years post-surgical treatment [11]. The tumor size, location, and some histological parameters have been identified as being involved in the recurrence of this neoplasm [11,20]. BCCs with the largest diameter, >10 cm (giant BCCs), have been associated with a higher risk of local recurrence [11,20]. Similarly, regarding the anatomic location, the H zone, i.e., the high risk of recurrence zone (the nose, orbital region, ears, and nasolabial folds) represents a relevant clinical parameter predictive of recurrence [11,20]. The presence of positive histological margins, as well as a vascular/perineural invasion and the infiltration of the reticular dermis and/or subcutis, are histological factors associated with BCCs with a higher risk of local relapse [11,20]. Moreover, the basosquamous, sclerosing/morphoeic, infiltrating, micronodular, and sarcomatoid BCCs are classically considered high-risk subtypes, while the nodular, superficial, pigmented, infundibulo-cystic, and fibroepithelial variants are considered low-risk BCCs [11,20]. Recently, Vornicescu et al. speculated about the possibility of identifying further immunohistochemical markers of BCCs that are associated with local recurrence in cases with histological tumor-free margins [11]. These authors evaluated the expression of the glioma-associated oncogene homolog 1 (GLI1), the yes-associated protein (YAP), the connective tissue growth factor (CTGF), and E-cadherin [11]. They found that a lower expression of CTGF may correlate with more biologically aggressive lesions, while no differences in the expressions of the remaining markers were observed in recurrent versus non-recurrent tumors [11]. 

The aim of the present study was to evaluate the potential association between the immunohistochemical expression of SRSF1 and the risk of the local recurrence of a series of 52 surgically excised BCCs with histological tumor-free margins (47 non-recurrent versus 5 recurrent tumors). In detail, we found high immunohistochemical levels of SRSF1 in only 30% of the non-recurrent tumors, while the remaining 70% exhibited a low SRSF1 immunohistochemical expression; conversely, a high SRSF1 immunoexpression was found in 80% of the recurrent BCCs from our cohort. Moreover, a statistically significant association (*p* = 0.0433) between high SRSF1 IRS and local recurrences were observed.

In addition, we also found significant differences between SRSF1 IRS values, the patient’s gender, the tumor anatomic site, and histological subtypes; higher and lower SRSF1 levels were exhibited by superficial and nodular BCCs, respectively. BCCs occurring at the extremities more frequently showed lower SRSF1 IRS. 

SRSF1 is a splicing factor that also plays a proto-oncogene role by stimulating angiogenesis, cell growth, and cell proliferation [21,22,23]. Its oncogenic role has been characterized in numerous human cancers [12,13,14,15,16,17,18,21,22,23]. It has been found to be downregulated in unaffected brain parenchyma, as compared to human glioblastoma tissue, where it appears to influence tumor angiogenesis by stimulating the formation of the proangiogenic form of the vascular endothelial growth factor A (VEGFA) [14]. The immunohistochemical expression of SRSF1 also correlated with a poor prognosis in adult diffuse gliomas, as well as with an increased cell proliferation in prostate cancer [17,18].

Our immunohistochemical results indicate a statistically significant correlation between higher levels of SRSF1 immunoexpression and an increased risk of the local recurrence of BCCs. Accordingly, we propose to add SRSF1 to the high-risk histological parameters of BCCs, even in the presence of safe tumor margins. Closer follow-up times might be necessary for those tumors which exhibit high levels of this protein. 

Although numerous high-risk clinical and histological parameters have been identified, the exact pathogenetic mechanisms underlying the local recurrence of BCCs, even in the presence of tumor-free surgical margins, is still to be elucidated. It has been hypothesized that YAP acts a key role for stem cell maintenance and BCC proliferation by negatively regulating the Hippo signaling pathway that has a tumor inhibition function [24,25]. If Hippo is not downregulated, YAP, along with other transcription factors, can stimulate the expression of protooncogenes with a final induction of cell proliferation, growth, epithelial-mesenchymal transition, and apoptosis [24,25]. We suggest that SRSF1, acting as a splicing factor/RNA binding protein, may selectively regulate the transcription of proteins involved in the YAP/Hippo pathway.

In addition, Mole et al. recently demonstrated in in vitro studies that the alternating cycles of phosphorylation/dephosphorylation may regulate SRSF1 expression and that the phosphorylation of the protein mediated by the human papillomavirus (HPV)-16 may lead to its cellular accumulation in differentiated HPV-affected keratinocytes [26]. These authors also showed that, while SRSF1 had an almost exclusive nuclear localization in undifferentiated HPV-affected keratinocytes, both the nuclear and cytoplasmic expressions were found in differentiated HPV-affected cells, speculating that HPV mediated a process of cytoplasmic transport and the accumulation of this protein during cell differentiation [26]. On the other hand, in the past decades, the relationship between HPV infection and BCC has been studied and well demonstrated. Zakrzewska et al. found that, although HPV infection was more frequent in cutaneous squamous cell carcinoma, beta-HPV DNA was present in about 55% of BCCs and, conversely, absent in the unaffected peritumoral skin [27]. Other authors reported statistically significant higher incidences of gamma-HPV infections in BCC patients [28]. The potential relationship between HPV infection and SRSF1 overexpression in BCCs is undoubtedly a suggestive hypothesis that deserves further investigation; however, we speculate that the exclusive nuclear localization of the protein found in the cases from our series contrasts with the data reported by Mole et al. [26], even if sometimes the results from in vitro and ex vivo studies can be discordant and the degree of differentiation of the keratinocyte lines used by the authors [26] does not overlap with that of BCC cells.

## 5. Conclusions

The present study suggests an active role of SRSF1 in inducing the local recurrence of BCCs; however, we emphasize that the expression of a single protein cannot be the only cause of a higher recurrence rate of BCC, but it must be inserted in a multifactorial process in which multiple factors play a complementary role. Further studies on a larger series of recurrent BCCs are required to confirm and validate our preliminary findings and to study the potential relationship between the overexpression of SRSF1 and other mechanisms potentially involved in BCC recurrence.

## Figures and Tables

**Table 1:** Clinico-pathological and immunohistochemical features of the BCCs from our series.

Number of Cases	Age Range	Gender	Anatomic Site	Local Recurrence	Histological Subtype	SRSF1 IRS
52	36–84 y	32 M; 20 F	Arms (*n* = 21) Legs (*n* = 15) Face (*n* = 10) Shoulders (*n* = 5) Ankles (*n* = 1)	No (*n* = 47) Yes (*n* = 5)	Nodular BCC (*n* = 27) Superficial BCC (*n* = 15) Sclerosing/morphoeic BCC (*n* = 6) Basosquamous carcinoma (*n* = 4)	L-IRS (*n* = 34) H-IRS (*n* = 18)

Abbreviations: y, years; M, male; F, female; BCC, basal cell carcinoma; L-IRS, low immunoreactivity score; H-IRS, high immunoreactivity score.

**Table 2:** Distribution of local recurrences according to SRSF1 immunoexpression.

Local Recurrence	High SRSF1 IRS	Low SRSF1 IRS
No	14	33
Yes	4	1

**Table 3:** Distribution of gender according to SRSF1 immunoexpression.

Gender	High SRSF1 IRS	Low SRSF1 IRS
Female	6	14
Male	12	20

**Table 4:** Distribution of tumor anatomic sites according to SRSF1 immunoexpression.

Anatomic Site	High SRSF1 IRS	Low SRSF1 IRS
Ankles	1	0
Arms	4	17
Face	5	5
Legs	4	11
Shoulders	4	1

**Table 5:** Distribution of histological subtypes according to SRSF1 immunoexpression.

Histological Subtype	High SRSF1 IRS	Low SRSF1 IRS
Basosquamous	2	2
Nodular	5	22
Sclerosing/morphoeic	2	4
Superficial	9	6

## Data Availability

All data presented in this study are available from the corresponding author upon reasonable request.
